# Health Literacy in Rural Areas of China: Hypertension Knowledge Survey

**DOI:** 10.3390/ijerph10031125

**Published:** 2013-03-18

**Authors:** Xia Li, Ning Ning, Yanhua Hao, Hong Sun, Lijun Gao, Mingli Jiao, Qunhong Wu, Hude Quan

**Affiliations:** 1 Department of Social Medicine, School of Public Health, Harbin Medical University, 157 Baojian Road, Nangang District, Harbin 150081, China; E-Mails: 363549688@qq.com (X.L.); ningninghyd@163.com (N.N.); hyhyjw@126.com (Y.H.); sunhong_1965@yahoo.com.cn (H.S.); gg73@163.com (L.G.); minglijiao@126.com (M.J; 2 Department of Community Health Sciences, University of Calgary, 3280 Hospital Dr NW, Calgary, Alberta T2N4Z6, Canada

**Keywords:** hypertension, knowledge, rural Chinese adult, hypertension education program

## Abstract

We conducted this study to determine levels and correlates of hypertension knowledge among rural Chinese adults, and to assess the association between knowledge levels and salty food consumption among hypertensive and non-hypertensive populations. This face-to-face cross sectional survey included 665 hypertensive and 854 non-hypertensive respondents in the rural areas of Heilongjiang province, China. Hypertension knowledge was assessed through a 10-item test; respondents received 10 points for each correct answer. Among respondents, the average hypertension knowledge score was 26 out of a maximum of 100 points for hypertensive and 20 for non-hypertensive respondents. Hypertension knowledge was associated with marital status, education, health status, periodically reading books, newspapers or other materials, history of blood pressure measurement, and attending hypertension educational sessions. Hypertension knowledge is extremely low in rural areas of China. Hypertension education programs should focus on marginal populations, such as individuals who are not married or illiterate to enhance their knowledge levels. Focusing on educational and literacy levels in conjunction with health education is important given illiteracy is still a prominent issue for the Chinese rural population.

## 1. Introduction

China is a large country with a population of about 1.339 billion, 50.3% of whom reside in rural areas [1] and has the largest hypertensive population in the World [2,3]. From 1991 to 2002, hypertension prevalence increased from 16.3% to 21.0% among urban adults, and from 11.1% to 18.0% among rural adults [2,4,5]. The increase in prevalence in rural areas is due to economic development, resulting in the adoption of urban lifestyles, as well as improved case-finding [6,7].

Hypertension-related diseases cost 31.89 billion Yuan Renminbi (RMB, approximately 4.8 billion dollars US) per year and result in about 11.4 years of life lost [8,9]. Chronic disease prevention has been identified as a high public health need in China’s 2009 health reforms, with national and local governments allocating 15 Yuan (approximately 2.2 dollars US) per person per year for basic public health services, including chronic disease prevention, with high priority placed on hypertension prevention and management [10]. In rural areas, primary care physicians are required to measure blood pressure, register and follow-up with patients, and provide hypertension education [11,12].

Rural areas in northeast China have the highest rates of hypertension and stroke in the country [6,7]. To eliminate the geographic disparity, efficient hypertension prevention, control, and management programs must be developed in these areas under the Chinese public health initiative. Health literacy is related to hypertension management, treatment and outcomes [13,14]. Andeseun *et al.* [14] surveyed 72 patients in newly initiated dialysis and found that the average reading of diastolic blood pressure was lower among patients with adequate health literacy skills than those without the skills. Thus, we conducted this study to evaluate the level of current hypertension knowledge, factors associated with hypertension knowledge, and the association between hypertension knowledge and the frequency of salty food consumption among hypertensive and non-hypertensive populations in rural areas of Heilongjiang province, China. Our findings are essential to identify the gaps in current hypertension knowledge, and thus to inform the development of effective health education programs for the prevention and management of hypertension.

## 2. Methods

### 2.1. Study Population

We conducted cross-sectional face-to-face surveys in hypertensive and non-hypertensive populations in the rural areas of Heilongjiang province, in the northeast of China. To obtain the two convenience samples, we first selected two counties (Fujin and Linkou), which were willing to cooperate with our survey and had convenient transportation access for our surveyors. Within each county, we categorized districts by economic development status (*i.e.*, relatively poor and non-poor, annual GDP ≥ 3,000 Yuan/person), and selected two districts from each category, for a total of eight districts. In each of the eight districts, villages with a minimum population of 800 were categorized into low, medium and high economic development groups, and one village was chosen from each group, for a total of 24 villages. The level of economic development was determined by county health department staff, who coordinated this survey, and were familiar with the economic status in the county.

This study included residents who were aged 30 years or older and resided in the village for at least five years. We excluded people who were incapable of participating in the survey due to mental or physical disorders, such as severe senile dementia and schizophrenia. Physicians in these villages register patients with hypertension. People without hypertension included in this study were those who were not recorded in the hypertension registry, were never diagnosed as hypertensive patients based on self-report, and had normal blood pressure measured at the time of this survey. A standardized mercury sphygmomanometer was used to measure blood pressure following the American Heart Association protocol [15], that is, performing three blood pressure measurements with the participant in the sitting position after five minutes of rest. In addition, participants were advised to avoid alcohol, cigarettes, coffee, tea, and exercise for at least 30 minutes before their blood pressure measurement.

### 2.2. Data Collection

Undergraduate medical students conducted face-to-face interviews from 23 to 26 July 2010, at village clinics and administration offices. Before the survey, the students were trained in survey administration and in blood pressure measurement, and had opportunities to practice interviewing. The survey, including questions on socio-demographic characteristics, health status, hypertension knowledge, attitude to health and disease prevention, and lifestyles, was tested and revised through a pilot study with 25 people (questionnaire available on request).

Physicians in the 24 villages were paid by fee-for-service plan and incentivized by the number of hypertensive patients who were registered and managed. Thus, the registry is relatively complete and frequently updated. We discussed our study population criteria with village physicians who registered hypertensive patients. These physicians reside in the villages and know the residents well. Nearly all the residents in the villages are farmers. We asked village physicians to invite all hypertensive patients and non-hypertensive residents in the 24 villages to participate in the survey. We attempted to survey the same number of hypertensive and non-hypertensive individuals in each village. Because of the convenience sampling strategy, the response rate was unknown, although the majority of hypertensive patients were likely surveyed.

Interviewers explained the purpose and confidentiality of the survey and then invited residents to participate; participation in the survey was accepted as oral consent. If there was more than one eligible person in a family, the first arrival to the interview site was interviewed. The completeness of questionnaires was checked immediately after the survey was administered and if there was missing information; individuals were resurveyed before they left the survey site.

### 2.3. Study Variables

Hypertension knowledge was assessed using an instrument which had been validated in a previous study [16]. The instrument contains 10 questions:

1. If someone’s blood pressure is 120/80, it is…(responses: high, low, normal, don’t know).
2. If someone’s blood pressure is 160/100, it is… (high, low, normal, don’t know).
3. High blood pressure can cause strokes... (yes, no, don’t know).
4. High blood pressure can cause heart attacks ... (yes, no, don’t know).
5. High blood pressure can cause kidney problems... (yes, no, don’t know).
6. High blood pressure can cause eye problems... (yes, no, don’t know).
7. Once someone has high blood pressure, it usually lasts for …(a few years, 5–10 years, the rest of their life, don’t know).
8. Losing weight usually makes blood pressure… (go up, go down, stay the same).
9. Eating less salt usually makes blood pressure… (go up, go down, stay the same).
10. People with high blood pressure should take their medicine… (everyday, at least a few times a week, only when they feel sick).

In addition, we collected information on socio-demographic characteristics (sex, age, education level, and marital status), self-perceived physical and mental health (5-point Likert-type scale of excellent, very good, good, fair, and poor), quality of life using the EuroQol-5 [17], which was translated into Chinese [18,19] , and the presence of 11 physician-diagnosed chronic diseases (*i.e.*, liver disease, lung disease, peptic ulcer disease, renal disease, arthritis, chronic back pain, diabetes, neurological disorder including stroke, cancer, allergy, and depression). Non physician-diagnosed chronic diseases were not included because the validity of self-diagnosed chronic disease depends on the level of the respondent’s knowledge, and their perceptions of “disease” and “health”.

Physician-diagnosed chronic disease was further confirmed by questions about the type of hospital where the diagnosis was made. Information was also collected on when blood pressure had been measured (within past 12 months, more than 12 months ago or never). Additionally we collected information on whether residents attended a hypertension educational session provided by clinicians and the provincial ministry of health in the last 6 months (yes, no), taken care of self health (yes, no), frequency of reading newspapers, magazines or books in the last month (at least once a week, less than once a week or never) and source of hypertension knowledge. Salty food consumption was defined as the frequency of eating salty pickled vegetables in the last week (none, 1–2 times, 3–4 times, 5–6 times and daily). In the region, salty pickled vegetables are very common as a side dish. Our survey was conducted in summer. Fresh vegetables are in abundance in summer and cost far less than in the winter season. Nearly all families store a large amount of salty pickled vegetables for winter. It was assumed that if respondents ate the salty pickled vegetable frequently in summer, they would eat it more frequently in winter. Thus determining the frequency of eating such a salty food is one way to assess the effectiveness of hypertension education programs in the region.

### 2.4. Statistical Analysis

Descriptive statistics were employed to describe characteristics of respondents with and without hypertension. A score of ten was assigned for each correct response, giving a maximum score of 100 per respondent. The hypertension knowledge score was found to be skewed and was normalized by the natural logarithm transformation. Multivariate linear regressions were used to determine factors associated with hypertension knowledge following a step-wise modeling strategy.

A linear regression model with hypertension knowledge as the dependent variable and one independent variable (such as age) was used to preliminarily assess a relationship between hypertension knowledge and the added independent variable. We repeated this modelling method by taking out the previously added variable and adding another independent variable until the association of the hypertension knowledge and each of the independent variables was examined. After this initial analysis, all independent variables were fitted by forward inclusion in a linear regression model to form a full or main effect model. Sequentially, one independent variable that was neither biologically meaningful nor statistically significant was removed from the full model. Then another insignificant independent variable was removed and previous variable was added to the model to observe changes of coefficient for each variable in the model to assess correlation among independent variables. We repeated this step until all variables were observed. Finally, independent variables that were not associated with hypertension knowledge (*p* < 0.05) were removed to form the parsimonious model.

Multivariate logistic regressions were used to assess responses to the question of “eating less salt usually makes blood pressure go down” (correct *versus* incorrect), and frequency of eating salty pickled vegetables in the last week (≥1 time *versus* less) and the association between hypertension knowledge and blood pressure targets (≥140/90 yes or no based on blood pressure measurements at the survey). In these two logistic regressions, all covariates collected in the survey were treated as potential confounding and adjusted.

## 3. Results

Of the 1,519 individuals who participated in the survey, 665 were hypertensive and 854 were non-hypertensive respondents (see Table 1). In both groups, a majority of the respondents were 50 to 64 years old, were married, and had a low level of education. Chronic disease was more frequent among hypertensive than among non-hypertensive respondents (71.6%*vs.* 54.8%). Quality of life scores were worse for hypertensives than non-hypertensive respondents.

**Table 1:** Characteristics of survey respondents.

Variable	Hypertensives	Non-hypertensives	*p*-value
	n (% of 665)	n (% of 854)	
Male	264 (39.7)	360 (42.4)	0.335
*Age (years)*			
30–49	96 (14.4)	349 (40.9)	<0.001
50–64	387 (58.2)	376 (44.0)	<0.001
≥65	182 (27.4)	129 (15.1)	<0.001
Married	570 (85.7)	772 (90.4)	0.005
*Education*			
Illiteracy	159 (23.9)	156 (18.3)	<0.001
Elementary school	355 (53.4)	376 (44.0)	<0.001
Junior high school or higher	151 (22.7)	322 (37.7)	<0.001
*Presence of any one of 11 chronic diseases* *	476 (71.6)	468 (54.8)	<0.001
*Quality of life (EuroQol-5)*			
Have walking problems	165 (24.8)	91 (10.7)	<0.001
Have washing and dressing problems	72 (10.8)	38 (4.4)	<0.001
Have problems with usual activities	153 (23.0)	81 (9.5)	<0.001
Have pain or discomfort	392 (58.9)	404 (47.3)	<0.001
Have anxiety or depression	336 (50.5)	341 (40.0)	<0.001
Self-perceived poor physical health	113 (17.0)	83 (9.7)	<0.001
Self-perceived poor mental health	79 (11.9)	59 (6.9)	<0.001
Not caring about self health	30 (4.5)	61 (7.1)	0.032
*Read books/newspaper/magazines at least once a week in the last month*	112 (16.8)	210 (24.6)	<0.001
*Attended hypertension educational session in the last 6 months*	318 (47.8)	288 (33.7)	<0.001
*Had blood pressure measurement*			
within 12 months	556 (83.6)	365 (42.7)	<0.001
≥12 months ago	109 (16.4)	52 (6.1)	<0.001
Never	-	437 (51.2)	-
*Systolic/diastolic blood pressure reading (mean)*	147/92	118/81	<0.001

* 11 chronic diseases included physician diagnosed liver disease, lung disease, peptic ulcer disease, renal disease, arthritis, chronic back-pain, diabetes, neurological disorder including stroke, cancer, allergy, and depression.

**Figure 1:**
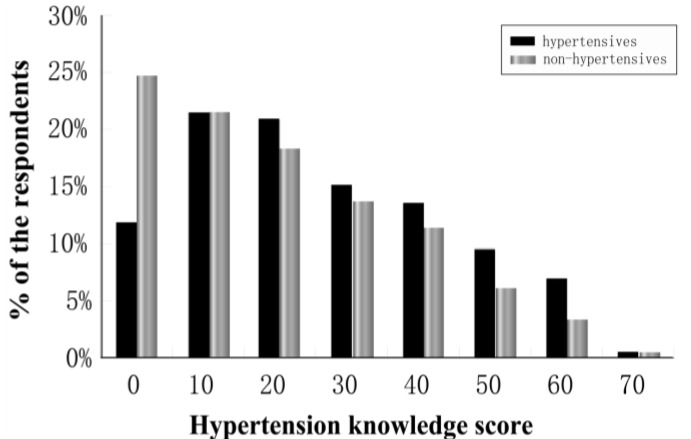
Percentage of the survey respondents by hypertension knowledge score (the maximum of 100).

The percentage of respondents with correct responses to the hypertension knowledge questions ranged from 18.0% to 71.9% among hypertensive respondents and 11.8% to 63.0% among non-hypertensive respondents (see Table 2). Of the respondents, 83.0% of hypertensive and 89.8% of non-hypertensive respondents had a score of fewer than 50 points out of a possible 100. Furthermore, 12.0% of hypertensive and 25.1% of non-hypertensive respondents had scores of 0, that is incorrect answers to all 10 questions (see Figure 1).

**Table 2:** Percentage of survey respondents with correct responses to hypertension knowledge questions.

Hypertension knowledge item	Hypertensives	Non-hypertensives	*p*-value
	n (% of 665)	n (% of 854)	
If someone’s blood pressure is 120/80, it is normal.	424 (63.8)	486 (56.9)	0.008
If someone’s blood pressure is 160/100, it is hypertension.	470 (70.7)	468 (54.8)	<0.001
High blood pressure can cause strokes.	243 (36.5)	268 (31.3)	0.04
High blood pressure can cause heart attacks.	259 (38.9)	274 (32.1)	0.006
High blood pressure can cause kidney problems.	120 (18.0)	101 (11.8)	0.003
High blood pressure can cause eye problems.	186 (27.9)	167 (19.6)	<0.001
Hypertension usually lasts for the rest of the life.	297 (44.7)	293 (34.3)	<0.001
Losing weight usually makes blood pressure go down.	356 (53.5)	443 (51.9)	0.596
Eating less salt usually makes blood pressure go down.	478 (71.9)	538 (63.0)	<0.001
People with high blood pressure should take their medicines everyday.	328 (49.3)	304 (35.6)	<0.001
Respondents with total score * < 50	552 (83.0)	767 (89.8)	<0.001
Respondents with total score * = 0	80 (12.0)	214 (25.1)	<0.001

* 10 points per correctly responded question item, the maximum of 100.

The average hypertension knowledge score was 25.6 out of the maximum 100 points for hypertensive and 20.0 for non-hypertensive respondents (see Table 3). Only a small proportion of respondents correctly answered questions about hypertension complications (*i.e.*, 36.5% for stroke, 38.9% for heart attack, 18.0% for kidney disease and 27.9% for eye disease among hypertensive respondents and 31.2% for stroke, 32.1% for heart attack, 11.8% for kidney disease and 19.6% for eye disease among non-hypertensive respondents). The average score varied by age, education and marital status.

**Table 3:** Average hypertension knowledge score (standard deviation, SD) out of the maximum 100.

	Hypertensives (N = 665)	Non-hypertensives (N = 854)	*p*-value
	Score (SD)	Score (SD)	
Overall	25.58 (17.78)	20.02 (17.40)	<0.001
*Sex*			
Male	25.61 (18.05)	20.94 (17.72)	<0.001
Female	25.56 (17.63)	19.35 (17.15)	<0.001
*Age*			<0.001
30–49 years	25.94 (17.08)	20.80 (16.38)	<0.001
50–64 years	26.93 (17.63)	20.61 (18.23)	<0.001
≥65	22.52 (18.18)	16.20 (17.24)	<0.001
*Education*			<0.001
Illiteracy	20.69 (17.43)	14.23 (15.70)	<0.001
Elementary schooling or more	27.11 (17.63)	21.32 (17.51)	<0.001
*Marital status*			<0.001
Married	26.19 (17.54)	20.49 (17.22)	<0.001
Others	21.89 (18.87)	15.61 (18.53)	<0.001

The factors associated with hypertension knowledge for both groups included marital status, education, when blood pressure had been measured, receiving hypertension education, and reading books, newspapers and magazines (see Table 4). In addition to these factors, mental health status was related to hypertension knowledge in non-hypertensive respondents (lower level for respondents with poor mental health) and the presence of a chronic disease was related to higher hypertension knowledge in hypertensive respondents. The major source of hypertension knowledge was health practitioners (62.4% for hypertensive and 32.2% for non-hypertensive respondents) and media (33.4% for hypertensive and 37.2% for non-hypertensive respondents).

**Table 4:** Factors associated with hypertension knowledge level among hypertensive and non-hypertensive respondents (risk adjusted coefficient and 95% confidential interval (95% CI)).

Variables	Full model *				Parsimonious model **	
	Hypertensives Coefficient P		Non-hypertensives Coefficient P		Hypertensives Coefficient (95% CI)	Non-hypertensives Coefficient (95% CI)
Male *vs.* female	0.015	0.703	0.027	0.431	-	-
Age (year)	−0.022	0.589	−0.019	0.616	-	-
Married *vs.* other	0.084	0.027	0.085	0.011	0.090 (0.012–0.115)	0.083(0.018–0.138)
Elementary schooling or more *vs.* illiteracy	0.114	0.004	0.107	0.003	0.119 (0.026–0.112)	0.114(0.034–0.128)
Measured blood pressure within 12 months *vs.* measured 12 months ago or never	0.155	<0.001	0.127	<0.001	0.166 (0.063–0.160)	0.095(0.016–0.089)
Have walking problems *vs.* no problem	−0.037	0.419	−0.053	0.179	-	-
Have washing and dressing problems *vs.* no problem	−0.012	0.794	0.073	0.096	-	-
Have problems with usual activities *vs.* no problem	−0.046	0.361	−0.039	0.385	-	-
Have pain or discomfort *vs.* no problem	0.053	0.192	−0.013	0.729	-	-
Have anxiety or depression *vs.* no problem	0.065	0.103	0.045	0.205	-	-
Not caring about self health *vs.* caring	−0.067	0.072	−0.020	0.545	-	-
Self-reported poor physical health *vs.* fair to excellent physical health	0.004	0.928	−0.029	0.472	-	-
Self-reported poor mental health *vs.* fair to excellent mental health	−0.011	0.819	−0.078	0.049	-	−0.10 (−0.179–−0.040)
Presence of any one of the 11chronic diseases **^†^** *vs.* none of them	0.117	0.002	0.044	0.218	0.129 (0.032–0.111)	-
Reading books, newspaper or magazines at least once a week in the last month *vs.* less frequently	0.075	0.047	0.152	0.000	0.078 (0.003–0.100)	0.164 (0.062–0.147)
Attended hypertension educational session in the last 6 months *vs.* none	0.150	0.000	0.067	0.050	0.158 (0.043–0.115)	0.079 (0.007–0.084)

* The model was fit using multivariate linear regression. Dependant variable is natural logarithm of the hypertension score (ln(score)) and independent variables included all variables listed in Table 1, such as sex, age, marital status, education, quality of life, chronic disease, self-reported mental and physical health, and caring about self-health. 95% CI = 95% confidence interval. ** The model included variables that were associated with hypertension knowledge. **^†^** 11 chronic diseases included physician diagnosed liver disease, lung disease, peptic ulcer disease, renal disease, arthritis, chronic back pain, diabetes, neurological disorder including stroke, cancer, allergy, and depression.

The frequency of eating salty pickled vegetables in the last week was lower among those who answered the question “eating less salt usually makes blood pressure go down” correctly than those who answered the question incorrectly (66.1% *versus* 70.1%, risk adjusted odds ratio: 0.834 and 95% confidence interval (95% CI): 0.578–1.202 among hypertensive respondents and 70.6% *versus* 77.2%, odds ratio: 0.710 and 95% CI: 0.514–0.979 among non-hypertensive respondents, see Table 5).

**Table 5:** Percentage and risk adjusted odds ratio (OR) * with its 95% confidence interval (95% CI) for eating salty pickled vegetable at least once in the last week by correct and incorrect response to the question “eating less salt usually makes blood pressure go down”.

Response to “eating less salt usually makes blood pressure go down”	N (%) of respondents eating salty picked vegetable ≥ 1 time in the last week	Risk adjusted OR (95% CI) for eating salty picked vegetable ≥ 1 time
*Among hypertensives*		
With correct response (n = 478)	316 (66.1% of 478)	0.83 (0.58–1.20)
With incorrect response (n = 187)	131 (70.1% of 187)	1.0
*Among non-hypertensives*		
With correct response (n = 538)	380 (70.6% of 538)	0.71 (0.51–0.98)
With incorrect response (n = 316)	244 (77.2% of 316)	1.0

Two logistic regression models were fit, one for hypertensives and another for non-hypertensives. Dependant variable is eating salty pickled vegetable at least once in the last week (yes or no); exposure variable is response to the question “eating less salt usually makes blood pressure go down” (correct or incorrect); other independent variables included variables listed in Table 1, such as sex, age, marital status, education, quality of life, chronic disease, self-reported mental and physical health, and caring about self-health.

Among hypertensive respondents, 48.3% had a systolic/diastolic blood pressure ≥140/90. The proportion slightly decreased with higher hypertension knowledge. However, the differences were not statistically significant after adjustment for covariates (see Table 6).

**Table 6:** Percentage and risk adjusted odds ratio (OR) * with its 95% confidence interval (95% CI) for systolic/diastolic blood pressure ≥ 140/90 among hypertensive respondents.

Knowledge Score	N (%) of respondents with blood pressure ≥ 140/90	Risk adjusted OR (95% CI)
(Maximum: 100)		For blood pressure ≥ 140/90
0	39 (48.8% of 80)	1.13 (0.68–1.87)
10	71 (48.6% of 143)	1.06 (0.72–1.57)
≥20	211 (47.7% of 442)	1.0

Dependant variable is blood pressure ≥ 140/90 at the time of survey (yes or no); exposure variable is hypertension knowledge level score based on the ten hypertension knowledge questions (10 score for each correct answer); We adjusted for variables listed in Table 1, such as sex, age, marital status, education, quality of life, chronic disease, self-reported mental and physical health, and caring about self-health.

## 4. Discussion

This survey clearly demonstrates that hypertension knowledge levels are extremely low in rural areas of northeast China. Although the level is statistically different between hypertensive and non-hypertensive respondents (mean score of 26 *versus* 20 out of a potential score of 100, *p* < 0.05), the difference is small. Many people lacked knowledge about hypertension complications and medication. Among non-hypertensive respondents, knowledge level was related to dietary behavior: people who knew about the relation between salty food and blood pressure ate salty food less frequently than those who did not know the relation.

Hypertension knowledge is extremely low, regardless of hypertension status, in our sample. Astonishingly, 12% of hypertensive and 25% of non-hypertensive respondents had a score of 0, incorrect answers to all 10 questions. The hypertension knowledge level in China rural areas we reported is much lower than previous reports from western countries [20,21,22,23,24,25,26,27]. For example, Sanne *et al.* [16] surveyed 296 hypertensive adults in the New Orleans metropolitan area using the same 10-item questionnaire as we used, and reported that 50% of the respondents answered at least seven questions correctly, and the correct response rate ranged from 41.9% to 98.0%, much higher than the 11.8%–63.0% range in our hypertensive respondents. Ayotta *et al.* [25] assessed hypertension knowledge using six questions among 1177 hypertensive patients in the United States. The study found correct response rates ranged from 43.9% to 93.1% across the six items, with 92.2% correctly answering the question of hypertension causing kidney problems, much higher than our finding of 18.0% on that item.

Our study demonstrated that hypertension knowledge levels were associated with marital status, education, health status, periodically reading books, newspapers or other materials, history of blood pressure measurement, and attending hypertension educational sessions provided by clinicians. However, the knowledge levels did not vary by age or sex. In our sample, 77.3% of hypertensive and 62.3% of non-hypertensive respondents were illiterate or had elementary schooling. Those who were illiterate had poorer hypertension knowledge than those with elementary or higher levels of schooling. This is congruent with our additional finding that people with regular reading habits had higher hypertension knowledge levels. These findings indicate that hypertension education programs should pay attention to adults with low literacy levels.

In fact, 62.4% of hypertensive and 32.2% of non-hypertensive respondents acknowledged that they received knowledge from their health practitioners. In the villages we studied, physicians or nurses regularly provide health education to residents, including hypertension education. We found that respondents who participated in the educational sessions had better hypertension knowledge than those who did not. In addition, hypertensive respondents with chronic diseases are likely to have better knowledge than those without, suggesting that healthcare providers take the opportunity to provide hypertension education when they have contact with their patients. Residents in rural areas generally do not go for regular health examinations unless they have a health problem, and thus have few opportunities to learn about hypertension.

Our study evidenced that current educational programs have positive effects. Respondents reported that village health practitioners are the major source of hypertension information and respondents who attended educational sessions had better knowledge than those who did not attend. Therefore, hypertension educational sessions should be continuously provided by village health practitioners, such as physicians and nurses, and should further target the marginal populations such as individuals who are not married, are illiterate or have poor mental health status. Since health practitioners live in the village, they are familiar with each individual’s social and cultural characteristics and health status and are therefore in a position to identify these target groups. In addition, village residents are likely to trust them, and therefore attend and follow the instructions provided in the educational sessions.

Non-hypertensive respondents who understood that salt was a risk factor for hypertension ate salty food less frequently than those who did not know that, illustrating that hypertension knowledge has an impact on preventive health behavior as reported by William *et al.* [20] There is an opportunity for educational programs to emphasize the long-term consequences of hypertension and to influence behavior. In this study, a majority of respondents were unaware of possible hypertension complications. For example, only 18% of the hypertensive and 11.8% of the non-hypertensive respondents knew hypertension could cause kidney disease. Only 49% of the hypertensive respondents knew that they needed to take medication daily. A lack of understanding of the long term health outcomes of hypertension may lead to poor compliance with antihypertensive medication; sufficient evidence indicates that patients have better drug-adherence if they are aware that an increased blood pressure could reduce their life expectancy [12,28]. Our study demonstrated that the proportion of hypertensive respondents who met blood pressure control targets (≥140/90) was slightly increased with higher levels of hypertension knowledge.

The survey found that respondents obtained hypertension knowledge mainly from the village clinics. Therefore, it is imperative to enhance roles of rural physicians in health literacy promotion. First, health professionals including nurses should be incentivized to lead inhealth promotion and education activities. Second, the Chinese government has invested resources for chronic disease management and control. A certain amount of these resources should be specifically allocated to hypertension. Third, blood pressure monitors should be installed at pharmacies and clinics for free measurement. Fourth, education program should target high risk populations and sodium reduction. The essential information about hypertension should be disseminated through radio, mobile phone text massage, and posters at the village clinics.

This study has limitations. First, we interviewed people who came to interview sites and were unable to assess an exact response rate. If this convenience sample had better health knowledge than those who were not surveyed, our findings about current hypertension knowledge levels could be over-estimated. Second, we missed people who were temporarily away from these villages for various reasons, such as working in urban areas. These people were deemed to be relatively healthy. Third, we surveyed two counties in the Heilongjiang Province of China; generalizing our findings to other regions should be done with caution. Fourth, we assessed salt consumption using frequency of eating salty pickled vegetable as a proxy. This method does not accurately quantify total salt consumption. Nevertheless, our study is consistent with previous studies in China [29,30], which reported low hypertension knowledge in rural areas.

Our study also has several strengths. The interview was conducted by medical students who are knowledgeable about hypertension and survey methods. The quality of data is likely reliable. Finally, hypertension was determined by a combination of blood pressure measurement, self-report, and a hypertension registry.

## 5. Conclusions

In conclusion, hypertension knowledge levels are alarmingly low in rural areas of China, particularly concerning hypertension complications and medication. Many factors contribute to this low hypertension knowledge level, such as the availability of health education programs, economic conditions, and cultural background. Promoting national education levels should be a national priority, because illiteracy is still a common issue for this population and must be overcome in order to improve health education. Knowledge content deficiencies that we identified could guide development and improvement of educational programs for rural populations with the goals of increasing the awareness of hypertension, promoting blood pressure monitoring, and actively managing the disease.
